# A Model-Driven Platform for Dynamic Partially Reconfigurable Architectures: A Case Study of a Watermarking System

**DOI:** 10.3390/mi14020481

**Published:** 2023-02-19

**Authors:** Roukaya Dalbouchi, Chiraz Trabelsi, Majdi Elhajji, Abdelkrim Zitouni

**Affiliations:** 1 Laboratory of Electronics and Microelectronics, University of Monastir, Monastir 5000, Tunisia; 2 Learning, Data and Robotics Lab, ESIEA Engineering School, 94200 Paris, France; 3 Electrical Engineering Department, College of Engineering, Shaqra University Dawadmi, Ar Riyadh 17441, Saudi Arabia; 4 Department of Physics, College of Science & Humanities, Imam Abdulrahman Bin Faisal University, Jubail 31961, Saudi Arabia

**Keywords:** reconfigurable architecture, dynamic partial reconfiguration, FPGA, UML/MARTE, automatic code generation, IP-XACT standard, video watermarking application

## Abstract

The reconfigurable feature of FPGAs (Field-Programmable Gate Arrays) has made them a very attractive solution for implementing adaptive systems-on-chip. However, this implies additional design tasks to handle system reconfiguration and control, which increases design complexity. To address this issue, this paper proposes a model-driven design flow that guides the designer through the description of the different elements of a reconfigurable system. It is based on high-level modeling using an extended version of the MARTE (Modeling and Analysis of Real-Time and Embedded systems) UML (Unified Modeling Language) profile. Both centralized and decentralized reconfiguration decision-making solutions are possible with the proposed flow, allowing it to adapt to various reconfigurable systems constraints. It also integrates the IP-XACT standard (standard for the description of electronic Intellectual Properties), allowing the designer to easily target different technologies and commercial FPGAs by reusing both high-level models and actual IP-XACT hardware components. At the end of the flow, the implementation code is generated automatically from the high-level models. The proposed design flow was validated through a reconfigurable video watermarking application as a case study. Experimental results showed that the generated system allowed a good trade-off between resource usage, power consumption, execution time, and image quality compared to static implementations. This hardware efficiency was achieved in a very short time thanks to the design acceleration and automation offered by model-driven engineering.

## 1. Introduction

Reconfigurable FPGA-based systems-on-chip (SoCs) can be modified several times after fabrication. Dynamic Partial Reconfiguration (DPR), supported by several FPGAs, offers the possibility to reconfigure some parts of the FPGA at runtime [1]. This allows for optimizing the system area by reusing the same hardware resources for different application tasks. However, it implies an increased system design complexity that can be seen at different design levels, such as designing reconfigurable application tasks, reconfigurable architecture regions, reconfiguration control, etc. Furthermore, other elements have to be taken into account by the designer, such as performance, resource usage, and power consumption. Therefore, there is a need for a platform that simplifies the designer’s work while meeting all the constraints of a reconfigurable design.

Model-Driven Engineering (MDE) can be a solution to reduce design complexity. UML (Unified Modeling Language) [2] is commonly used in this approach for system modeling. The graphical nature of this language facilitates system understanding, editing, and reuse. UML profiles are a way to apply this language, originally used for software modeling, to other domains such as embedded systems. A UML profile is a set of extensions of the basic UML elements, which we call stereotypes. These stereotypes are used to describe different features of a given domain. There are several standard UML profiles for embedded systems, such as SysML (System Modeling Language) and MARTE (Modeling and Analysis of Real-Time and Embedded systems) [2].

One of the advantages of using MDE is that it allows for generating a system implementation code from high-level models. To allow this feature, MDE approaches often use a deployment phase that links the components of high-level models to low-level implementations through IPs (Intellectual Properties). All the necessary code for IP communication and system implementation is then generated automatically from high-level models. Furthermore, the code of some high-level components, with non-existent implementing IPs, can be generated from the UML descriptions of their behaviors, such as UML state machines. This can be the case with the reconfiguration controller, for instance. The main issue of the deployment phase of most MDE methodologies in the literature is that the deployed hardware IPs are tightly linked to the implementation technology, which makes both high-level models and actual IPs hardly reusable for various technologies. In this context, a standard representation of the system components is required to enhance design reuse.

The IP-XACT specification developed by the SPIRIT consortium offers a standard XML description of IP meta-data independent of the used language. This facilitates system integration using compatible descriptions from multiple component vendors and allows IPs to be easily reused and exchanged between electronic design automation (EDA) tools for SoCs [3]. Therefore, this standard has been increasingly used lately in FPGA tools. In the context of model-driven designs, IP-XACT can be used as an intermediate representation to go from high-level models to executable code.

The progress in FPGA technologies has enabled them to embed a growing number of computing resources on one chip, targeting increasingly sophisticated applications. This has led to a more complex design handling different aspects related to runtime adaptation. In this context, it may be difficult to analyze all the control data in a centralized way. Designing a decentralized control by splitting the control problem between autonomous controllers, each handling the self-adaptation of a reconfigurable component, can be beneficial for complex reconfigurable systems. A coordinator that has visibility only on global system requirements and constraints can be used in this approach, leaving local control data management to the distributed controllers. This reduces the complexity of the controllers, facilitating their verification and their reuse as well as the scalability of the control model. Therefore, the design flow proposed in this paper offers the possibility to design both centralized and decentralized control, depending on the requirements of the control problem handled by the target system.

This paper proposes an MDE-based design flow and platform for dynamically reconfigurable SoCs aiming at reducing design complexity through the following contributions:

- High abstraction level modeling and automatic generation of the implementation code without the need for in-depth knowledge of many low-level aspects of the FPGA technology. An extended version of the MARTE profile is used to integrate reconfiguration-modeling concepts. The separation of concerns is used when designing the models (application, architecture, etc.) to promote their reuse.
- Flexible control decision-making, handling both centralized and decentralized solutions that are easily reusable and scalable for various reconfiguration control requirements.
- Transforming high-level architectural models into IP-XACT files, thus targeting different tools supporting this standard.
- Using abstract metamodels and model transformations to be easily adaptable to designs targeting other reconfigurable FPGA technologies.
- Generating FPGA scripts, in addition to the automatic generation of C/VHDL code, so the synthesis and implementation flow can be run automatically to obtain bitstreams ready to be loaded onto the target FPGA.
- Accelerating design exploration (according to the functional and non-functional constraints defined at the beginning of the design process) thanks to the automation of model-driven engineering.

The proposed design flow was validated through a reconfigurable video watermarking application as a case study. Experimental results showed that the generated watermarking system allowed a good trade-off between resource usage, power consumption, execution time, and image quality compared to static implementations. 

The rest of the paper is organized as follows: Section 2 gives a short review of the related work in the field. Section 3 illustrates the proposed design flow and platform. Section 4 presents the case study video watermarking application. Section 5 describes the use of the proposed design flow for this application, and Section 6 discusses the experimental results. A list of the abbreviations, variables, and control parameters used in this paper is available in Appendix A. A list of the used software and hardware systems is available in Appendix B.

## 2. Related Work

SoCs are composite and complex systems that can include several hardware and software IPs and various processors for software tasks. Thanks to their reconfigurability, FPGA devices extend the concept of SoCs, making them able to adapt to various changes in user preferences, application requirements, and standards [4]. However, this implies additional design tasks to handle system reconfiguration and control, which increases design complexity [5]. To address this issue, several approaches have been proposed based on MDE tools.

In the literature, many works have proposed high-level modeling to reduce the design complexity of DPR SoCs. In this context, several works used MDE to automatically generate the code necessary for the system implementation from high-level models, thus reducing design complexity and time-to-market. For example, the authors in [6] presented a development framework based on UML/MARTE models for dynamically configurable systems design. In this framework, an XML-based tool was used to generate a VHDL and SystemC code from high-level models. In addition, the work in [7] proposed a design methodology based on UML/MARTE to deal with DPR designs. In this work, Xilinx concepts were included in the high-level models, which limits its flexibility. In [8], the authors proposed a UML/MARTE framework aimed at designing and synthesizing a formal controller managing dynamic reconfiguration. The UML/MARTE description of the formal controller was transformed into a BZR language in [9] to build a correct-by-construction executable controller; the controller code is generated in C language and can then be added to a reconfigurable FPGA design to be executed by the processor. However, this work dealt only with the modeling and generation of the controller and did not cover the high-level modeling of the rest of the reconfigurable system. One of the main issues of most MDE approaches for reconfigurable systems design in the literature is that they end with a deployment phase that is still tightly linked to the implementation technology. This leads to generating system IPs that are dependent on a given technology, which makes the high-level models for these IPs hardly reusable for various technologies. Therefore, a standard representation of the system components is needed to enhance the design reuse for various FPGA tools and vendors. Indeed, using a standard representation of the system components in high-level modeling facilitates the reuse of both models and actual IPs for different targets.

The IP-XACT standard, offering an XML description of the system IPs independently of the used language, is a good solution to face the SoCs design reuse challenge. Some works proposed the integration of IP-XACT and UML in MDE-based design since they are complementary tools for the information they deliver [10]. Indeed, IP-XACT is a standard for IP exchange and also a great solution that promotes IP reuse. In the literature, the exploitation of both specifications has been used in a variety of works to generate IP-XACT design files from MARTE models. This approach was proposed in the context of static SoC designs [11] as well as for reconfigurable ones. In [12,13], the authors proposed an approach that has some similarities with ours. This approach is based on the transformation of MARTE models into IP-XACT descriptions to automatically generate reconfigurable systems code. The main limit of this work is that it is tightly linked to the implementation technology (Xilinx Tools) and does not provide a metamodel that is abstract enough to easily target other technologies such as Intel FPGAs. Furthermore, the reconfiguration control design in this work was very simplistic since the reconfigurations were launched according to a system mode selected by the user. Our design flow offers a reconfigurability control design that can easily adapt to various application requirements. It also presents a higher control design flexibility, enabling both centralized and decentralized reconfiguration control.

## 3. Proposed Design Flow

The proposed design flow is called the generic design flow for dynamic partially reconfigurable systems (GDF4DPR). The term generic is explained by the fact that even though not many FPGA vendors provide dynamic reconfiguration, this is a tendency that will cover all FPGA vendors and our design flow also aims to be used for future reconfigurable FPGAs.

**Figure 1:**
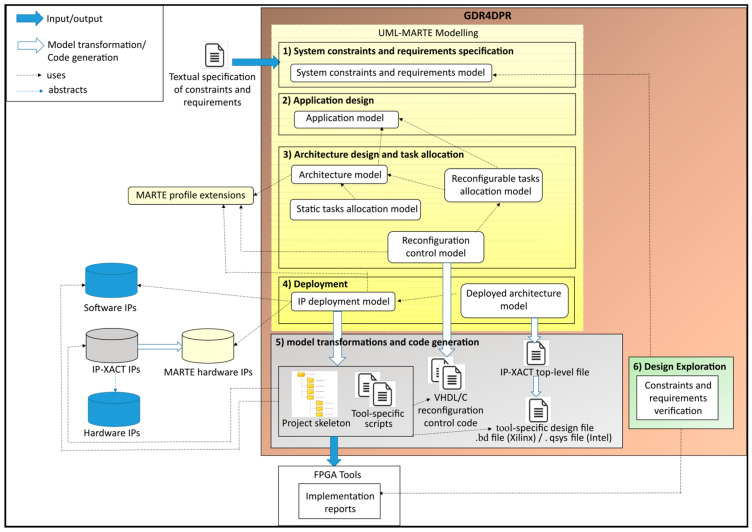
The proposed GDF4DPR design flow.

The flow is based on six steps, where the first four steps imply high-level modeling covering: (1) system constraints and requirements specification, (2) application design, (3) architecture and task allocation design, and (4) deployment modeling. The fifth step is model transformations and code generation, and the sixth one is design exploration, as illustrated in Figure 1.

The proposed model-driven design platform was implemented using the Eclipse framework using plugins for high-level modeling, model-to-model transformations, and model-to-text transformations. After realizing the high-level modeling (the first four steps), the designer launches the transformation process, which generates all the system code (in VHDL and C languages). It also generates tool-specific scripts and a tool-specific design file. The designer can then launch the FPGA design tool (such as Vivado for Xilinx FPGAs), and execute the generated tool-specific scripts, which automates synthesis, implementation, and bitstreams generation. During the design exploration step (step 6), designers verify, using FPGA design tools, if the generated system respects the functional and non-functional constraints and requirements specified at the beginning of the design flow. If not, high-level models can be edited, and code can be regenerated automatically. A list of the software and hardware systems used in this work is available in Appendix B.

Thanks to the concern separation of the first four steps, the used models can be easily modified and reused for different system specifications or to target different FPGA platforms. In this section, the design flow is described through the used UML/MARTE concepts and the extensions we propose for the MARTE profile. The application of these concepts and extensions is presented in Section 5 with a watermarking case study.

### 3.1. System Constraints and Requirements Specification

This represents a critical step that directly affects the final system. Indeed, at this stage, the designer must include all the useful information to analyze the need of the system. This information groups together the system requirements, the user requirements, the constraints to be respected, etc. This information can be related to performance, power consumption, quality of service, etc. It is mainly modeled using the <<nfpConstraint>> MARTE stereotype (non-functional constraints), as will be illustrated in Section 5 with the watermarking case study. These requirements and constraints will be then verified during design space exploration (step 6) to converge towards an implementation that makes a good trade-off between constraints and requirements.

### 3.2. The Application Design

In the second step of the flow, the application is described as a set of interconnected tasks, each handling a specific function. This stage is thus dedicated to the modeling of the application tasks and the communication between them using mainly UML/MARTE components, ports, and connectors. Depending on the needs of the target system, the application tasks can be physically implemented in software or hardware. This choice is made in the next step.

### 3.3. Architecture Design and Task Allocation

**Figure 2:**
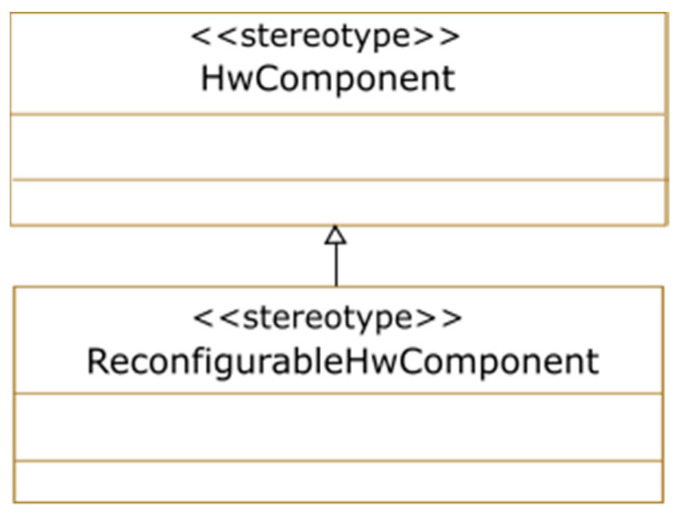
Reconfigurable hardware component stereotype.

At this stage, the static and reconfigurable parts of the system are modeled using UML/MARTE components, ports, and connectors. To model the reconfigurable part of the architecture, we extended the MARTE profile through the <<ReconfigurableHwComponent>> stereotype, which inherits from the <<HwComponent>> MARTE stereotype as shown in Figure 2. Using this new stereotype, we can distinguish components that will be implemented statically from those that will be reconfigurable.

This stage also specifies which tasks will be implemented in software by the processor(s) and which tasks will be implemented in hardware using the FPGA logic. Task allocation on processors or static hardware components is performed using the <<allocate>> MARTE stereotype. 

As for reconfigurable tasks, we propose to use the <<mode>> and <<configuration>> concepts of MARTE together with the <<allocate>> stereotype. Depending on the system requirements, reconfiguration control can be either centralized or decentralized, in which case each reconfigurable region is handled by a separate module. In both cases, state machines are used for reconfiguration decision-making, where each state (<<Mode>>) represents a different configuration of one or several reconfigurable regions. To model these state machines, the <<modeBehavior>>, <<mode>>, and <<modeTransition>> MARTE stereotypes are applied, respectively, to UML state machines, states, and transitions. The MARTE <<configuration>> stereotype is used here to describe the task allocation on reconfigurable regions. Our GDF4DPR design flow offers two ways of modeling <<configurations>>. First, a <<configuration>> can model task allocation on a single reconfigurable region for a given <<Mode>>. In this case, a separate state machine is used to model each region, so a <<configurations>> describes task allocation on this region for a given <<Mode>> of the state machine. In the second case, a <<configuration>> models task allocation on multiple reconfigurable regions. In this case, the behavior of reconfigurable regions can be modeled either by a separate state machine for each region or by using a single state machine to model the different modes of all the reconfigurable regions of the system, so a <<configurations>> describes, in the second case, either a combination of the active modes of all the state machines of the reconfigurable regions or an active global mode in a global state machine.

**Figure 3:**
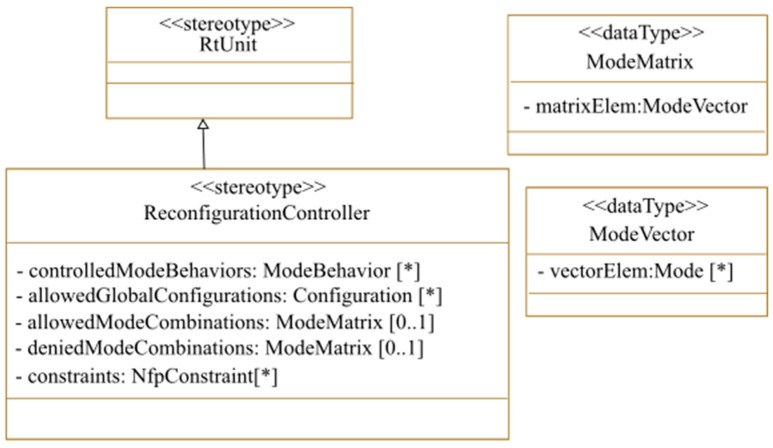
Reconfiguration controller stereotype (* UML Syntax that means zero or more).

In both centralized and decentralized reconfiguration decision-making, a global controller is required to guarantee that the reconfiguration decisions respect the global system requirements. To model this controller, we propose the <<ReconfigurationController>> stereotype as an extension to the <<RtUnit>> (Real-Time Unit) MARTE stereotype. As shown in Figure 3, we offer various options for the reconfiguration controller modeling. First, the designers can mention the allowed global system configurations, which are already modeled using the <<configuration>> stereotype. This option is used when each MARTE <<configuration>> describes the active modes of all the reconfigurable regions. For example, for a system having two reconfigurable regions and three possible modes for each region, if we use four different MARTE <<configurations>>, modeling each the active mode of the two reconfigurable regions, the allowedGlobalConfiguration field of the <<ReconfigurationController>> will take these four configurations. The second way to coordinate reconfigurable regions’ active modes is to use the allowedModeCombinations field of the <<ReconfigurationController>>. Each allowed mode combination is a combination between the active modes of all the reconfigurable regions. This option is useful when the designer separately models a MARTE <<configuration>> for each reconfigurable region active mode. Likewise, designers can specify the denied mode combinations, so that when a distributed reconfiguration decision is made targeting a denied mode combination, this reconfiguration is not launched. Non-functional constraints can also be attached to the controller to be considered for reconfiguration decision-making using the constraints field. Static and reconfigurable tasks allocation, as well as reconfiguration control modeling, will be illustrated later with an example in the watermarking case study.

### 3.4. Deployment

**Figure 4:**
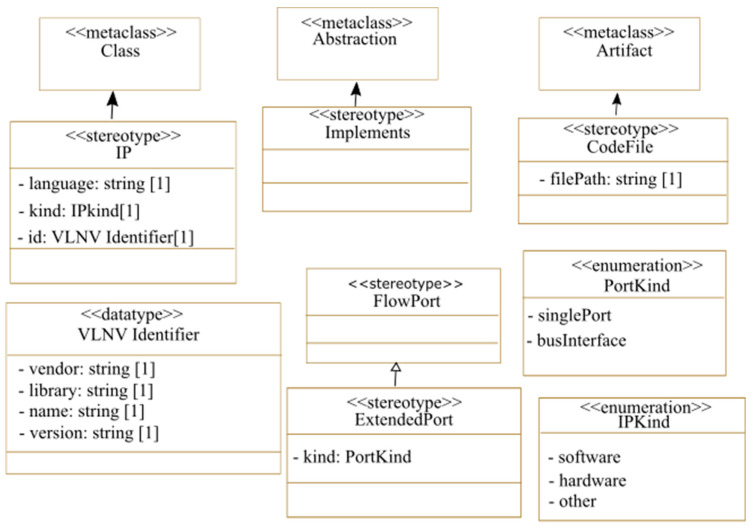
Proposed extensions to the MARTE profile for IP deployment.

At this stage, the implementation details of the application tasks and the architecture components are specified through IPs. To be used in the deployment model, IPs have to be already packaged according to the IP-XACT standard and then transformed into MARTE components and stored in a MARTE components library. Figure 4 describes the extensions we propose to the MARTE profile for the deployment modeling. The <<IP>> stereotype can be applied to MARTE components representing either software IPs to be executed by the processor or hardware IPs to be implemented on the FPGA logic. Designers specify the language of the IP, its type (software or hardware), and its VLNV (vendor, library, name, and version) identifier. This identifier references a unique IP-XACT component in the library, which is used when transforming the whole high-level system into an IP-XACT global design. At the deployment stage, the <<CodeFile>> stereotype is used to attach code files to IPs. To link hardware and software modules to the implementing IPs, we propose the <<implements>> stereotype. The communication between IPs is then described by modeling their ports and linking them. At this stage, the designer has to distinguish single ports from bus interfaces. This information is important for generating the IP-XACT global design. To model this, we propose the <<ExtendedPort>> stereotype as shown in Figure 4. This stereotype offers two kinds of ports. The single-port kind is used to specify a point-to-point connection and the bus interface kind is used for a bus connection.

Once the software and hardware IPs are specified in the deployment model, model transformations are used to become closer to the implementation code as detailed in the next section.

### 3.5. Model Transformations and Code Generation

#### 3.5.1. MARTE Deployed Architecture to IP-XACT Design

An IP-XACT design file describing the whole architecture is generated from the deployed architecture model and is based on a set of transformation rules: Every instance of a MARTE component having the <<IP>> stereotype is transformed into a “component instance” according to the IP-XACT standard. Connections between the MARTE components in the deployed architecture are then transformed into either IP-XACT interconnections, adHocConnections, or hierConnections depending on the type of the connected ports (single port or bus interface) and the levels of hierarchy between the connected components.

#### 3.5.2. IP-XACT Design to Tool-Specific Design

**Figure 5:**
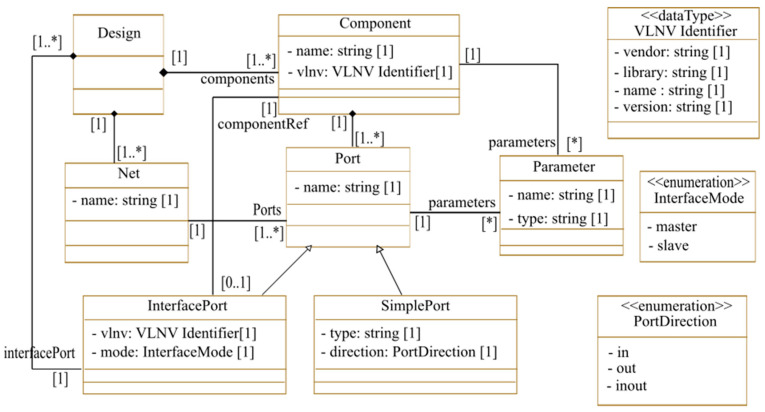
*Xilinx* Block Design eta-model (* UML Syntax that means zero or more).

After transforming the MARTE deployed architecture into an IP-XACT design, the latter is transformed into a tool-specific design to be imported into an FPGA tool. To implement the system using the Vivado tool for Xilinx FPGAs, the generated tool-specific design can be in form of a .bd (Block Design) file. For Intel FPGAs, a .qsys file can be generated to implement the system using the platform designer tool. To make this transformation easily reusable and extendable for different tools and technologies, we used a model-to-model transformation before generating the tool-specific design file. We defined a metamodel for the tool-specific design. The IP-XACT design file is first transformed into a model corresponding to the defined metamodel. Finally, we translated the obtained model into code. Figure 5 shows the used Xilinx Block Design metamodel. In this metamodel, a « Design » represents the main platform element which contains a set of interconnected components. Each block design is made up of a set of components linked together by simple ports, nets, or interfaces. Each component is defined with its VLNV identifier which is specified in its IP-XACT description. Ports in the Block Design can be either simple ports, which correspond to simple ports in IP-XACT, or Interface ports, which correspond to interfaces in IP-XACT. Nets in the Block Design correspond to connections in the IP-XACT design. The obtained Block Design model is then translated into a .bd file. Indeed, through a few modifications, the Block Design metamodel for Xilinx tools can be reused to describe another tool-specific design, such as the Intel Platform Designer .qsys design file.

#### 3.5.3. Generation of the Reconfiguration Control Code

This step corresponds to the generation of the code necessary for reconfiguration decision-making and reconfiguration launching. It consists in translating MARTE <<modeBehaviors>> (state machines) into code for reconfiguration decision-making together with the necessary code to launch reconfiguration. For this, we propose two methods. The first one is purely software-based where decision-making and reconfiguration launching is performed by a processor. The second one is mixed, where MARTE <<modeBehaviors>> are translated into VHDL state machines. These machines are coordinated by the processor which handles also reconfiguration launching. This step will be detailed later in the case study section.

#### 3.5.4. Generation of the Processor Code

The high-level model describing task allocation on the processor is translated into a processor code containing function calls to the software IP linked to these tasks at the deployment phase. This code also contains the code necessary for reconfiguration decision-making and reconfiguration launching.

#### 3.5.5. Generation of Tool-Specific Scripts and Project Skeleton

At this step, the proposed platform creates a project skeleton in which it copies: (1) the hardware and software IPs codes specified at the deployment phase of the high-level modeling, (2) the generated tool-specific design file, (3) the generated hardware code for reconfiguration control, and (4) the generated code for the processor. Tcl scripts are also generated at this phase so the synthesis and implementation flow can be run automatically to obtain bitstreams ready to be loaded onto the target FPGA. The generated scripts handle the following steps: (1) importing the generated design file, (2) launching Synthesis for the static part of the system and reconfigurable modules, and (3) loading the Synthesis results in the floor planning tool. Designers can then place the reconfigurable regions as desired. This is the only task that is performed manually since we cannot predict the resource usage of the reconfigurable regions before synthesizing them and therefore their placement is not completed in the high-level models. The rest of the generated scripts are then launched to automatically implement different configurations of the system and to generate the related bitstreams.

### 3.6. Design Exploration

The objective of this step is to verify that the generated system respects the functional and non-functional constraints and requirements specified at the beginning of the design flow. Different kinds of verifications can be completed at this stage, such as functional verification, through simulation or during runtime execution, resource usage and timing reports verification using FPGA tools, power consumption estimation/measuring, etc. Thanks to these verifications, the designer can make the necessary corrections and modifications in the high-level model of the concerned design phase to respect the system requirements and constraints. The system code can be then regenerated automatically and re-verified, if necessary, which significantly accelerates the exploration phase.

In the next section, we describe the video watermarking application that we will use as a case study to validate the proposed design flow for reconfigurable systems.

## 4. Case Study: A Video Watermarking Application

### 4.1. Overview

In this section, we use a video watermarking application to validate the proposed design platform. This application is intended for copyright protection to protect the video sequence from hacking and illegal use. The watermarking technique consists of inserting a watermark in a multimedia document (image, text, audio, and video) to identify the author of the document while respecting the security and the visual quality that represent the major requirements of the user. We call a watermark [14], the information message inserted in the multimedia documents. The choice of the message content is arbitrary, it can be a card number, binary sequence, image, audio, etc. In this work, we used a binary sequence inserted in an invisible way as a watermark to guarantee the robustness of the algorithm. In addition to the watermark, it is necessary to determine the watermark insertion space, such as the frames I, B, and P, the DCT coefficient, motion vector, phase angle, etc. In this application, we used as insertion space the vertical and horizontal components of the even motion vectors. We exploited the method presented by Y. Dai [15] in which the residual motion is used as insertion space. The same suggestion is used in [16], where the insertion of the watermark depends on the type of phase angle of the motion vectors. Several reasons justify the choice of motion vectors. Motion vectors are unaffected by non-geometrical distortions such as noise, color corrections, and digital filters. In addition, the motion is independent of alterations caused by analog-digital conversion and intra-frame compression of videos [17]. Furthermore, our aim is not only to identify the author but also to ensure overall video protection. For this reason, we included the scrambling technique in the application. This technique [18] is greatly exploited in radio and television broadcasting to protect the broadcast data. In this video application, this technique consists in swapping the motion vectors. The permutation order of the vectors is given later as a key delivered only to the authorized users to access the video sequence. 

From a watermarking system implementation point of view, our work presents some novelties. First, the architecture that we use in this paper is the only FPGA-based implementation using motion vectors for watermark insertion. Furthermore, to the best of our knowledge, it is the first dynamic partially reconfigurable FPGA-based architecture for a video watermarking system.

### 4.2. Application Tasks

**Figure 6:**
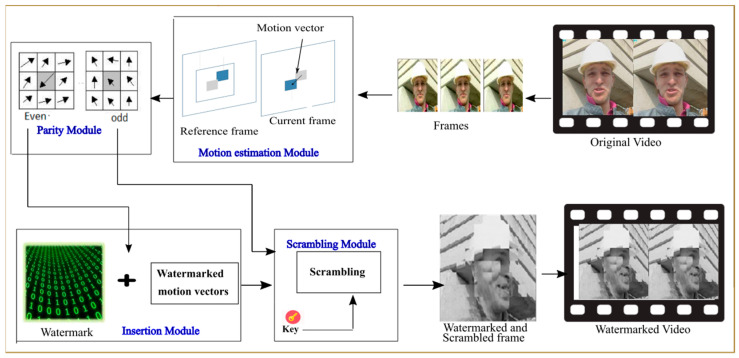
Overview of the watermarking application.

The suggested application is composed of five main modules which are the motion estimation, the sum, the parity, the insertion, and the scrambling module, as shown in Figure 6. In this work, to maintain the robustness of the architecture and satisfy the invisibility constraint of the watermarking application, we used an invisible insertion method. The insertion of the watermark in the even motion vectors is carried out following several steps. The first step is to extract the video frames. Then, for every frame, the motion vectors are generated and classified into two groups: the first group contains the even motion vectors and the second contains the odd motion vectors. The watermark to be inserted is represented by a sequence of binary values. Only a single bit of the watermark is inserted into the vertical and horizontal axis of the even motion vectors, given that the number of inserted bits equals the number of even motion vectors eventually generated. Then, all generated vectors (even and odd) from each frame are scrambled. Once the motion vectors are scrambled, the vector permutation order is subsequently exploited as a security key assigned to the authorized users. The watermarking application is performed in the compressed domain in which the embedding and extraction of the watermark do not need complete decoding and re-encoding of the compressed video versus the uncompressed domain, which implies a reduction in computational overhead [19]. 

### 4.3. Adaptive Motion Estimation

In the proposed architecture, the motion vectors represent the insertion space of the watermark. These vectors are generated in the motion estimator (ME) module, which is the most critical component in video compression. Its purpose is to minimize the temporal redundancy between the successive frames of the video. This has been used by various video-coding standards such as H.265 and H.264. The H.265, also known as standard High-Efficiency Video Coding (HEVC), is the most used in a wide range of new multimedia applications [20]. The basic goals and objectives of this standard are upgrading the coding efficiency in comparison with the H.264/AVC standard by decreasing the bit rate for equal video quality [21]. 

In this case study, we use a motion-adaptive watermarking system using two block-matching algorithms to generate the motion vectors, the gradient search (GS) and the four-step search (4SS). Depending on the movement speed in the video, the estimator will implement either the GS or the 4SS algorithm. The objective of reconfiguration here is to have a good trade-off between resource usage, power consumption, execution time, and PSNR (Peak Signal-to-Noise Ratio). Indeed, previous works showed that, for slow video sequences, the GS algorithm produces a higher PSNR than the 4SS one, though at the cost of a significantly higher execution time. On the other hand, for fast video sequences, these algorithms offer close PSNRs. This makes the 4SS algorithm more suitable for this type of video due to its accelerated execution. Both block-matching algorithms are implemented using nine Processing Elements (PE) running in parallel to generate the SAD (sum of absolute differences) value. In the case of slow movement, the estimator will be reconfigured by the nine PE of the GS algorithm or else by the nine PE of the 4SS algorithm. In our previous works [22,23], we proposed a software/hardware implementation of a video watermarking architecture. The architecture was dedicated to the fast video sequence where we used the 4SS algorithm. 

Generally, there are three types of objects in a video sequence: objects that keep the same motion for certain frames and other objects that move in an almost constant way between two sequential frames. The third type is objects moving in slow or fast motion in the same frame [24]. In this application, the reconfiguration is based on the recognition of the type of movements of each video frame and then choosing the suitable algorithm that must be activated during the execution time. The SAD can reflect very well the degree of motion activity in the video sequence having different movement activities. As will be shown later, the estimator module used in this case study integrates an adaptation module that makes reconfiguration decisions according to the motion activity. This module acquires the average SAD generated by the estimator as input. Subsequently, this average is compared to several thresholds to choose between the GS and the 4SS algorithm and launch reconfigurations if needed.

## 5. Design Flow Validation

Video watermarking is a real-time application that requires high processing capacities. Indeed, a small delay in video stream processing can lead to high degradation in the visual quality. This application can thus benefit significantly from the hardware acceleration offered by FPGA. The change of the motion estimation algorithm according to the video motion speed makes the video watermarking application well-adapted to dynamic partial reconfiguration.

### 5.1. System Constraints and Requirements Specification

**Figure 7:**
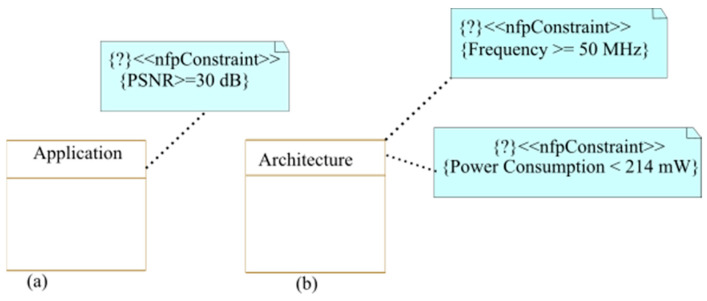
System specification modeling: (**a**): application constraints and (**b**): architectural constraints.

As the first step of high-level modeling, the designer specifies the requirements and constraints of the system. To model constraints linked to non-functional properties, we used the *<<nfpConstraint>>* MARTE stereotype as presented in Figure 7. To determine the requirements imposed by a video watermarking application and architecture, we carried out a study to extract the PSNR [25] values expected in video watermarking applications. The power consumption and frequency constraint were defined regarding a previous implementation using the motion estimator module proposed in [26]. This implementation was an adaptive non-DPR (non-Partial Dynamic Reconfiguration) one. The system contained, at the same time, hardware modules implementing both the GS and the 4SS algorithms. Only one of these algorithms was active at a given time depending on the motion speed. In this case study, we aimed at reducing the power consumption compared to this work thanks to resource usage reduction using DPR.

### 5.2. The Application Design

**Figure 8:**
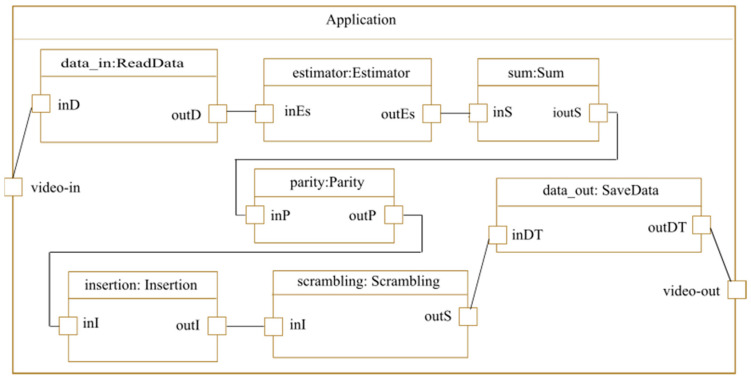
Application modeling.

The application phase consists in modeling the different application tasks and the interconnections between them. In this phase, the tasks are defined abstractly independently of the used technology, which makes the application model easily reusable for other system configurations. The modeling of the case study application is presented in Figure 8. It contains seven tasks (five tasks are specific to the case study application and two are reusable tasks). The reusable tasks allow for reading and displaying data before and after processing (watermarking and scrambling). The ports of each application task are stereotyped as «FlowPort». This MARTE stereotype is used to indicate the direction of each port (in, out, or in/out) to describe the data flow direction. After receiving the video frames using the «ReadData» task, these data are then transmitted to the «Estimator» task for the calculation of the SAD value. Once the motion vectors are generated, we use the «Sum» and «Parity» tasks, respectively, to calculate the sum of the motion vectors and find the even motion vectors for watermark insertion. The insertion of 1 bit of the watermark in the vertical and horizontal components of the even motion vectors is performed by the «Insertion» task. Finally, all motion vectors are scrambled using the «Scrambling» task and the data are saved using the «SaveData» task.

In this case study, we use two motion estimation algorithms (GS and 4SS described earlier) according to the motion speed, so the Estimator task will be implemented with two algorithms as will be specified in the rest of the GDF4DPR design flow.

### 5.3. Architecture Design and Task Allocation

**Figure 9:**
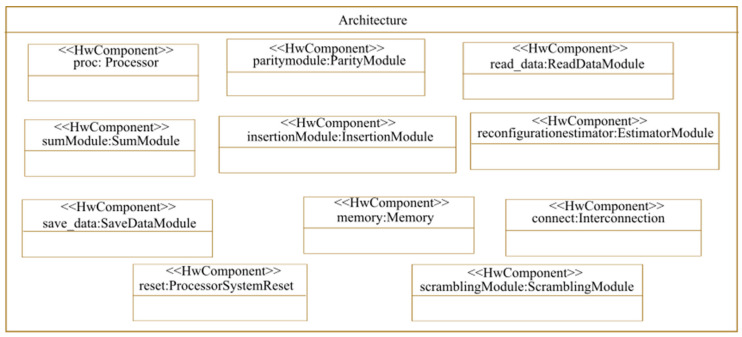
Architecture modeling.

In this phase, we modeled the architecture as presented in Figure 9. The IPs of the proposed architecture are stereotyped as «hwComponent». We modeled the components of the architecture abstractly and independently of the used technology. That is why we did not specify the name and the number of ports of each IP. Since we used a hardware implementation of the watermarking application, the architecture contains a hardware component for each application task. The architecture also contains a storage component and a processor. In addition, we used the IP «Interconnection» to ensure the communication between the IPs of the architecture and the IP«ProcessorSystemReset» for processor reset.

**Figure 10:**
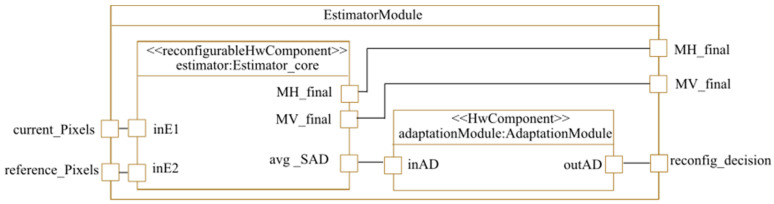
Estimator modeling.

Once the architecture components are specified, the designer must detail the reconfigurable part of this architecture. As mentioned above, the motion estimator used will implement either the GS or the 4SS algorithm at a given time. The operation of one algorithm or the other depends on the video motion speed. Each PE is implemented in a reconfigurable region. As shown in Figure 10, the estimator module contains an estimator core that implements the nine PEs that will be reconfigured with either the GS or the 4SS algorithm at runtime according to the video motion speed. To indicate that the estimator core will be implemented in a reconfigurable region, we added the <<reconfigurableHwComponent>> stereotype to the MARTE profile. The estimator core has as inputs the current and the reference pixels and provides as results the horizontal and the vertical components of motion vectors (MH_final and MV_final). The estimator module also contains an adaptation component that we added so that reconfiguration decision-making (using a state machine) is completed in the hardware and then communicated to the processor to launch reconfigurations.

After describing the static and reconfigurable parts of the architecture, the allocation of the application tasks on the architecture component is modeled. The allocation in the MARTE profile consists in attaching each abstract component of the application to its execution support in the architecture. The designer performs two types of allocation. The first type is the allocation of static tasks, which will be implemented in the static part of the system, and the second type is the allocation of reconfigurable tasks, which will be implemented in the reconfigurable regions.

#### 5.3.1. Allocation of the Static Tasks

**Figure 11:**
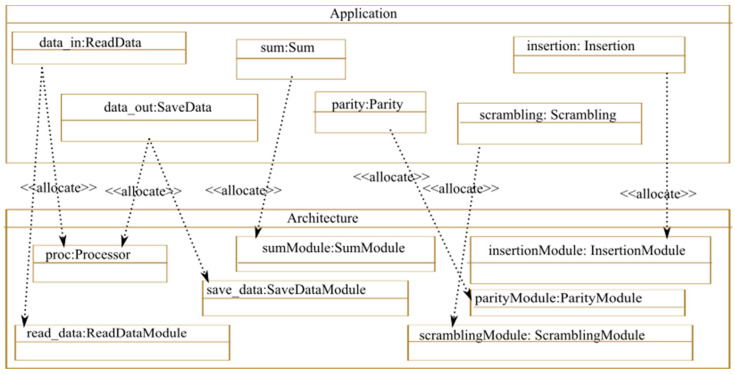
Static task allocation.

In this step, the task allocation of the processor and static hardware components is achieved using the <<allocate>> MARTE stereotype. As shown in Figure 11, in our example, the processor manages the reading and saving of data. The rest of the application tasks are implemented in the hardware and are allocated to the corresponding hardware components of the architecture, except for the estimator module since it is a reconfigurable module.

#### 5.3.2. Allocation of the Reconfigurable Tasks and Reconfiguration Control Modeling

As mentioned above, both centralized and decentralized reconfiguration decision-making approaches are possible in the proposed GDF4DPR design flow. For this case study, since all the PEs implemented in the reconfigurable regions have to be reconfigured at the same time with the same content, centralized decision-making is used here. In case the target system has reconfigurable regions handling various tasks, allocating a separate controller to each task allows it to make reconfiguration decisions related to the reconfigurable region. These decisions can be either authorized or denied later by a global coordinator to respect global system requirements and constraints. In this case, a separate state machine would be used for each reconfigurable region. The decentralized control would have many advantages in this case, such as decomposing the control design complexity between controllers, providing easier access to local data for decision-making, and enabling easier placement and routing of distributed controllers compared to a centralized hardware controller. 

**Figure 12:**
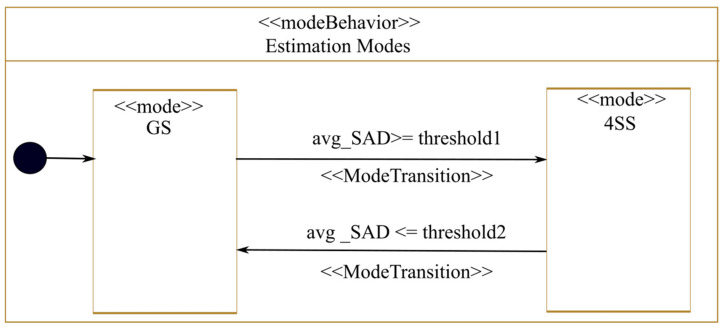
Reconfiguration controller modes.

In our case study, we modeled one state machine that represents the behavior of all reconfigurable regions using the <<modeBehavior>> and <<mode>> MARTE stereotypes as shown in Figure 12. The reconfigurations conditions are attached to the state machine transitions. The «ModeTransition» of the MARTE profile makes it possible to determine the transition from a source mode to a target mode. Since we decided to implement reconfiguration decision-making in the hardware, this state machine is linked to the adaptation module contained in the global estimator component as described earlier. As shown in Figure 12, the adaptation module will receive the average SAD (avg_SAD) value obtained after performing motion estimation on a whole frame as input. Based on this value, the adaptation module will deduce if the estimator is currently handling a slow or a fast sequence. The GS algorithm is used initially. After processing a frame, the estimator sends the average SAD to the adaptation module. If this value exceeds a SAD threshold1, the adaptation module deduces that it is a fast video sequence and then requests a reconfiguration that loads the 4SS algorithm. If the current configuration is the 4SS algorithm and the average SAD value is below a second threshold, threshold2, the adaptation module deduces that it is a slow video sequence and then requests a reconfiguration that loads the GS algorithm. The SAD thresholds used in this work are threshold1 = 600 and threshold2 = 400. These thresholds were extracted from the work in [27], which is based on a study of different video sequences of 300 frames.

**Figure 13:**
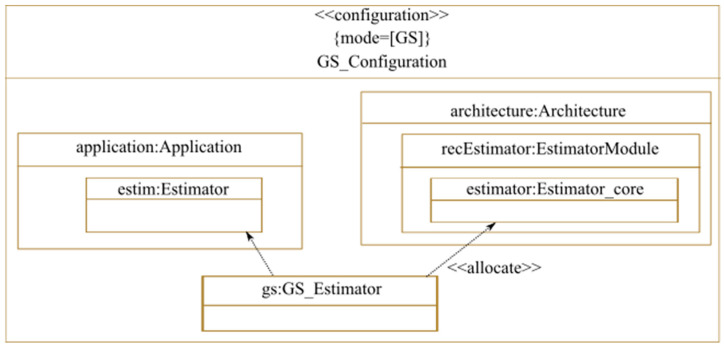
Allocation of the reconfigurable estimator task (the GS mode).

In this case study, the reconfigurable region performs two modes of the same application task. Therefore, it is necessary to indicate the version of the task to be allocated to a region for each configuration of the reconfigurable region. Figure 13 represents a MARTE configuration used to model one of the possible configurations (modes) of the reconfigurable estimator, which is the GS mode. Therefore, the GS version of the estimator task of the application is allocated to the estimator core module contained in the “EstimatorModule” of the architecture. 

**Figure 14:**
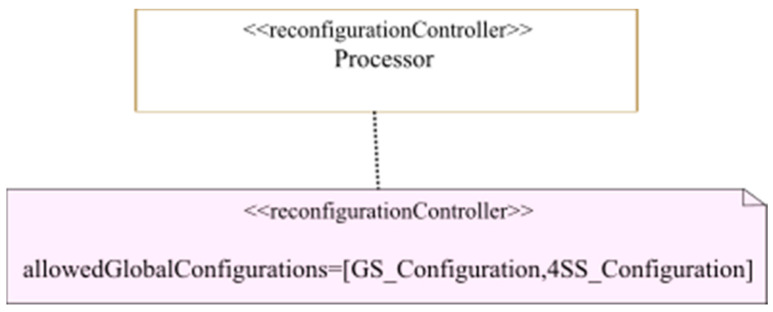
Reconfiguration controller.

As we mentioned earlier, in both centralized and decentralized reconfiguration decision-making, a global controller is required to guarantee that reconfiguration decisions respect the global system requirements. In this case study, this task is performed by the processor. To do so, we apply the <<reconfigurationController>> stereotype, described earlier in Section 3.3, on the processor as shown in Figure 14. Here, we use the allowedGlobalConfigurations field, which indicates that the allowed global system configurations are the GS_Configuration modeled in Figure 13 and the 4SS_Configuration. This corresponds to the two system configurations where all the nine reconfigurable regions implement one of the two used estimator algorithms. In case a decentralized control is used, coordination is performed between several state machines related to separate controllers. In this case, we can use other fields of the <<ReconfigurationController>> stereotype. As described earlier in Section 3.3, the allowedModeCombinations and deniedModeCombinations fields of the <<ReconfigurationController>> can be used when the designer models a MARTE <<configuration>> separately for each reconfigurable region active mode. The mode combination in this case is a combination of the active modes of all the reconfigurable regions. The coordinator can thus either allow or deny a reconfiguration decision made by a controller if it would lead to a mode combination that is not among the ones specified in the allowedModeCombinations field or is rather specified in the denied mode combination. Non-functional constraints can also be attached to the coordinator to be taken into account for reconfiguration decision-making, using the constraints field of the <<ReconfigurationController>> stereotype.

### 5.4. Deployment

**Figure 15:**
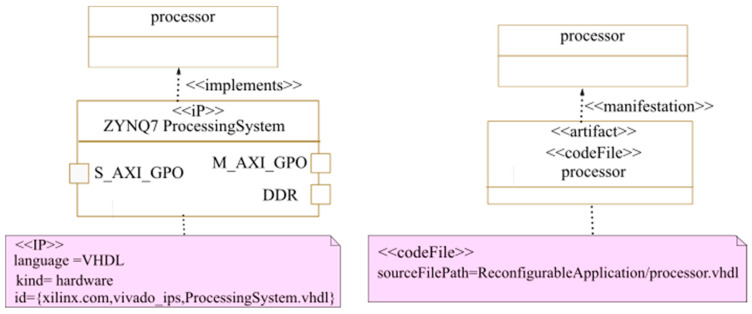
IPs deployment: example of the processor IP.

The deployment phase consists in assigning to each elementary component of the architecture or application a software or hardware IP implementing it. We use the <<implements>> stereotype to link the IP, which has the <<IP>> stereotype, to the application task that it implements. The code files of the used IPs are also specified at this stage using the <<codeFile>> stereotype. As shown earlier in Figure 4, the <<IP>>, <<implements>>, and <<codeFile>> stereotypes are extensions we propose to the MARTE profile. The hardware IPs used at the deployment phase are retrieved from a MARTE components library obtained from a transformation of IP-XACT-compliant IPs. Thanks to the use of the IP-XACT standard, the IPs can be easily integrated and reused in various FPGA vendor tools. In addition to the application hardware IPs, the designer must specify IPs for other architectural elements, such as the processor and the interconnect. Figure 15 shows an example of the deployment of a technology-dependent IP. In this case study, the Zynq Processing system implements the processor for this application since we target a Zynq FPGA.

**Figure 16:**
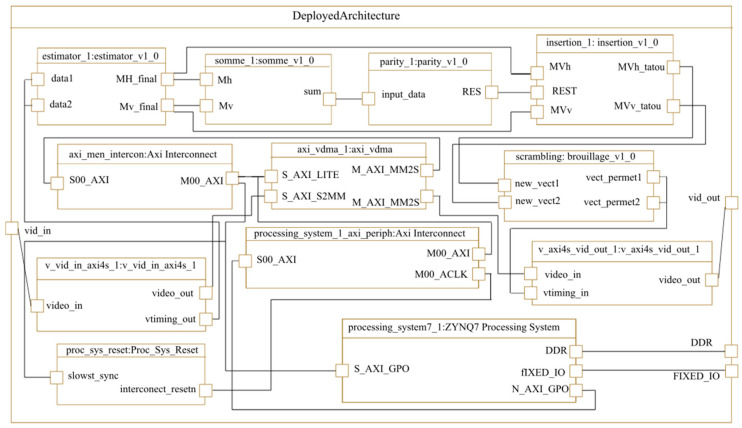
The deployed architecture.

The last step of high-level modeling is the description of the deployed architecture. This step deals with the interconnections between the architecture IPs as shown in Figure 16.

### 5.5. Model Transformations and Code Generation

At this stage, the deployed architecture is transformed into an IP_XACT design file, where MARTE components having the <<IP>> stereotype are transformed into an IP-XACT “component instance”. Connections between the components of the deployed architecture are then transformed into either IP-XACT interconnections, adHocConnections, or hierConnections depending on the type of the connected ports (single port or bus interface) and the levels of hierarchy between the connected components. The obtained IP-XACT design file is then transformed into a tool-specific design file. In this case study, the IP-XACT file was transformed to obtain a .db (Block Design) file to be used in the Xilinx Vivado tool. To make this transformation easily reusable and extendable for different tools and technologies, we used model-to-model transformation before generating the tool-specific design file.

In addition to the generation of the tool-specific design file, our platform generates the reconfiguration control code. For this case study, the modeled state machine modeled in Figure 12 was transformed into a VHDL code to implement the “adaptation module” of the estimator modeled in Figure 10. As for reconfiguration launching, our platform generates C code for the processor to execute. In this code, the processor reads a register from the estimator where the adaptation module has written the number of the configuration to be loaded. The processor then launches the required reconfiguration. In the case of multiple reconfiguration decision-making state machines (it is not the case in this case study), a coordination code is added to the processor where it can accept or refuse to launch a reconfiguration required by a given adaptation module. 

The final step of code generation is to generate tool-specific Tcl scripts, so all the synthesis and implementation flow can be run automatically to obtain bitstreams ready to be loaded onto the target FPGA. Our design flow generates three scripts. The first one handles the following steps: (1) importing the generated design file, (2) launching Synthesis for the static part of the system and reconfigurable modules, and (3) loading the Synthesis results into the floor planning tool. Designers can then place the reconfigurable regions using the floor planning tool. This is the only task that is performed manually since we cannot predict the resource usage of the reconfigurable regions before synthesizing them and therefore their placement is not completed in the high-level models. In this application, the reconfigurable region size corresponds to the size of PEs of the 4SS algorithm since it requires more resources than the GS algorithm. The second script is then launched to automatically implement different configurations of the system and to generate the related bitstreams. The last script compiles the software to be executed by the on-chip processor and generates the whole system image to be loaded on FPGA.

### 5.6. Design Exploration

After the first generated script is launched, we can check the consistency of our architecture before launching the second script that implements the system configurations. At this phase, we can verify that the static part, comprising interfaces to the reconfigurable regions, is consistent for all the configurations. For this, we use a design rule checker, which allows for detecting and managing errors in a report form. After launching the second script, we can detect if the reconfigurable regions were optimally placed and sized. Designers can easily replace these regions if necessary to have better resource usage results. Other verifications are also possible through simulation or at runtime. If the detected errors or problems are related to the structure of the application, the architecture, tasks allocation, or components deployment, this can be solved by modifying the high-level models and regenerating the system code automatically. This makes design exploration faster and spares the designer from tedious, error-prone, and long development cycles.

## 6. Experimental Results and Discussion

In a previous work [23], we implemented the watermarking application on a static FPGA-based system (only the 4SS algorithm was used for motion estimation); this implementation did not use a model-driven approach. Using the GDF4DPR design flow proposed in the present paper, we noticed a significant design acceleration thanks to the automatic generation of the whole system code, as well as tool-specific files and scripts, from high-level models.

The proposed reconfigurable watermarking system aims to make a good trade-off between the following requirements: resource usage, power consumption, execution time, and PSNR for various motion speeds. Therefore, in the rest of this section, we present the implementation results of the reconfigurable watermarking system in terms of resource usage, power consumption, and reconfiguration time. We also compare execution time and PSNR results to those of static implementations.

### 6.1. Resource Usage

In the proposed architecture, we use nine reconfigurable regions. In each reconfigurable region, a PE implementing either the GS or the 4SS algorithm is loaded, depending on the generated SAD average value. The reconfigurable region size is determined based on the largest reconfigurable module (RM) size to be loaded, which in our case is the RM implementing the PE with the 4SS algorithm. The hardware resources used by an RM implementing the 4SS algorithm are summarized in Table 1.

The resource usage of the whole system was compared to three static versions of the system: a static version implementing only the GS algorithm, a static version implementing only the 4SS algorithm, and a third version using adaptive motion estimation proposed in our previous work [26]. The difference between the third version and the one used in the current paper is that it did not use dynamic partial reconfiguration. The estimator in [26] contained hardware modules implementing both the GS and the 4SS algorithms present at the same time on the chip, but only one of these algorithms was active at a given time depending on the motion speed. Despite its adaptivity to motion speed compared to an estimator implementing only one algorithm, this solution has a non-negligible resource usage overhead as shown in Table 2. The resource usage of the dynamic partially reconfigurable architecture is significantly lower than the one used in [26] since only one algorithm is present on the board at a given time. If we compare the resource usage of the DPR version with the static versions implementing only one estimation algorithm, we notice that DPR implies a slight resource usage increase, which is due to the additional hardware modules needed for reconfiguration (reconfiguration decision-making and reconfiguration interface).

### 6.2. Reconfiguration Time

As for bitstreams, the GDF4DPR design flow generated one full bitstream and a set of partial bitstreams (nine partial bitstreams for the PEs of the GS algorithm and nine for the 4SS algorithm). The full bitstream is the one to be loaded initially and it contains the static part of the system and the partial bitstreams of the GS algorithm. Using SAD values, the adaptation module of the estimator decides which algorithm has to be executed and sends an interruption to the processor if a reconfiguration is required. The processor then launches the required partial reconfiguration through the PCAP (Processor Configuration Access Port) interface. The 32-bit PCAP interface has a theoretical reconfiguration speed of 400 MB/s when clocked at 100 MHz on a Xilinx Zynq SoC. The total size of the nine partial bitstreams is 1595 Kb, which implies a reconfiguration time of 30 ms. This reconfiguration time is not negligible. This is because the total size of the reconfigurable module is quite high. Since the reconfiguration is based on two thresholds applied to the average SAD produced after processing each frame, this implies a small number of reconfigurations. This is because successive frames have, most of the time, similar average SAD values, unless the type of the motion changes significantly, which we manage using the SAD thresholds.

### 6.3. Power Consumption

Our watermarking system was tested using three video sequences of 300 frames in the QCIF format. These sequences have three different motion behaviors: slow, medium, and fast. The average power consumption of the reconfigurable watermarking system while processing the three video sequences was 140 mW. The same video sequences were executed in the non-DPR version of the watermarking system using the adaptive motion estimator proposed in [26]. The average power consumption of the system was 214 mW, which shows that the DPR version allowed a power consumption reduction of 34.57%. Compared to the static versions implementing only one estimation algorithm, we notice that the DPR implies a slight increase in power consumption (up to 5%), which is due to higher resource usage and runtime reconfiguration.

### 6.4. Performances

Since one of the objectives of using two different motion estimation algorithms is to have a good trade-off between execution time and video quality (represented by the PSNR), we compared the PSNR and processing time given by our DPR system with the same three non-DPR versions described earlier: the static version implementing only the GS algorithm, the static version implementing only the 4SS algorithm, and the version using adaptive motion estimation proposed in our previous work [26] (alternating both algorithms depending on motion speed). The experimental results are described in Table 3. The execution times presented in Table 3 are the average execution times per frame for three different motion-speed video sequences. For our DPR version, the reconfiguration time is included in the average execution time per frame.

As shown in Table 3, for the slow sequence (Akiyo), the DPR version requires a slightly higher execution time than the static GS version. This is due to the slight overhead of the reconfiguration time. On the other hand, the DPR version provides better PSNR results. If we compare the DPR version with the static 4SS version, we notice that the DPR version provides better PSNR results, but at the same time, a significant difference in execution time. This is because the SAD thresholds used in the DPR version make it prefer the usage of the GS algorithm for most frames in this video sequence since it provides better PSNR results than 4SS for slow video sequences. Compared to the version in [26], the DPR version provides better PSNR results but a longer execution time due to the reconfiguration overhead.

As for the medium-motion sequence (Foreman), the DPR version provides better PSNR results than the other systems. It also has a faster execution time than the GS version. This is because this video sequence has more fast-motion frames than slow-motion ones, depending on the used SAD thresholds, which makes the DPR version apply the 4SS algorithm more than the GS one for this video sequence. Compared to the version in [26], the DPR version provides a slightly better PSNR and faster execution time.

For the fast sequence (Silent), the DPR version provides better PSNR results than the other systems. Its execution time is between the GS and the 4SS execution times since it applies the 4SS algorithm more than the GS one for this video sequence. This is because, for fast sequences, the 4SS algorithm has faster execution times than the GS one with comparable PSNR results. Compared to the version in [26], the DPR version provides a better PSNR and faster execution time.

The implementation results described in this section show that the reconfigurable watermarking architecture generated by the proposed design flow makes a good trade-off between execution time, video quality, resource usage, and power consumption. The proposed model-driven approach shows a significant design acceleration thanks to the high-level modeling and the automatic generation of VHDL/C code and tool-specific files and scripts.

## 7. Conclusions

In this paper, we propose a model-driven design flow and platform for dynamic partially reconfigurable architectures aiming at accelerating the design of reconfigurable systems to be implemented on FPGAs by reducing design complexity, automating code generation, and facilitating design reuse. The GDF4DPR is based on high-level modeling that covers several design steps from system specification to physical implementation. As for reconfiguration decision-making modeling, the proposed design flow handles both centralized and decentralized control solutions to adapt to various reconfigurable systems constraints. It proposes an automatic transformation of high-level models into IP-XACT designs to ensure IP reuse and to target different technologies and commercial FPGAs. The proposed flow enables the automatic code generation of the whole system, including tool-specific files and scripts, to increase designer productivity. As a case study, we used a video watermarking application. 

It can be concluded that the proposed flow supports all the design stages of dynamically reconfigurable architectures. Its high-level modeling allows the designer to easily target different technologies. The implementation results show that the proposed platform allowed us to automatically generate a dynamic partially reconfigurable watermarking architecture that provides a good trade-off between adaptivity, performance, resource usage, and power consumption. In future work, we intend to enhance the proposed platform to support more complex reconfigurable systems and offer more automatic generation possibilities.

Although the tool-specific design files generated by the design flow are currently targeting Xilinx FPGAs, we designed the tool-related metamodel and model transformation in a way that makes them easily reusable and extendable to target different reconfigurable FPGA technologies, which is one of the future directions of our work. In the presented case study, the performance/energy consumption trade-off was handled by fixing SAD thresholds to minimize the reconfiguration number and save energy. In future work, we intend to integrate more sophisticated power/performance optimization techniques into the proposed design flow to target various reconfigurable systems constraints. Dynamic task partitioning is also an interesting feature to add to the proposed design flow to cover more requirements of reconfigurable systems. 

## Figures and Tables

**Table 1:** Resource usage of a reconfigurable PE.

Resources	Resource Usage of a PE Implementing the 4SS Algorithm
slice	96
LUT	75
LUT as memory	0
RAMB10	0

**Table 2:** Comparison between the static and reconfigurable watermarking systems in terms of resources.

Architectures	Resources			
	Resource Usage in LUT (Out of 53,200)	Resource Usage in LUTRAM (Out of 17,400)	Resource Usage in FF (Out of 106,400)	Resource Usage in IO (Out of 200)
The static version using only GS	13,229	29	2887	67
The static version using only 4SS	12,267	32	1878	29
The adaptive non-DPR version proposed in [26]	25,648	48	4852	142
The DPR version	4500	235	5748	62

**Table 3:** Comparison between the static and reconfigurable watermarking systems in terms of performance.

Architectures	Performances					
	Akiyo		Foreman		Silent	
	Execution Time (ms)/Frame	PSNR	Execution Time (ms)/Frame	PSNR	Execution Time (ms)/Frame	PSNR
The static version using only GS	1.5681	43.120	1.92456	31.500	1.63944	35.411
The static version using only 4SS	0.85536	42.977	0.85536	32.076	0.9266	35.660
The adaptive non-DPR version proposed in [26]	1.140048	44.286	1.85328	32.010	1.35432	35.120
The DPR version	1.620584	44.83	1.72958	32.60	1.19792	35.78

## Data Availability

Not applicable.

## Appendix A

**Table A1:** Abbreviations list.

Abbreviation	Explanation
4SS	Four-Step Search
Bd	Block Design
DCT	Discrete Cosine Transform
DPR	Dynamic Partial Reconfiguration
EDA	Electronic Design Automation
FPGA	Field-Programmable Gate Array
GDF4DPR	Generic Design Flow for Dynamic Partially Reconfigurable systems
GS	Gradient Search
H.264/AVC	Advanced Video Coding
HEVC	High-Efficiency Video Coding
IP	Intellectual Property
IP-XACT	XML format that describes the meta-data of IP designs and flows and the interconnection of IP interfaces in a standard specification
LUT	Lookup Table
MARTE	Modelling and Analysis of Real-Time and Embedded systems
MDE	Model-Driven Engineering
ME	Motion Estimator
NoC	Network-on-Chip
PCAP	Processor Configuration Access Port
PE	Processing Elements
PSNR	Peak Signal-to-Noise Ratio
QCIF	Quarter Common Intermediate Format
RtUnit	Real-Time Unit
SAD	Sum of Absolute Differences
SoC	Systems-on-Chip
SPIRIT	Sharing Partnership for Innovative Research in Translation
SysML	System Modelling Language
Tcl	Tool Command Language
UML	Unified Modelling Language
VHDL	Very High-Speed Integrated Circuit Hardware Description Language
VLNV	Vendor, Library, Name and Version
XML	Extensible Markup Language

**Table A2:** Variables and control parameters list.

Variables and Control Parameters	Explanation
MV_final	Vertical components of motion vectors
MH_final	Horizontal components of motion
Avg_SAD	average SAD
Threshold1	First SAD threshold
Threshold2	Second SAD threshold

## Appendix B

**Table A3:** A list of used software and hardware systems.

Component	Details
High-level modelling and code generation software	Eclipse IDE for Java Developers: Version: Luna Service Release 1a (4.4.1)
FPGA design tool	Vivado v2019.2
FPGA device	Zynq-7000
